# Vascular Integrity and Signaling Determining Brain Development, Network Excitability, and Epileptogenesis

**DOI:** 10.3389/fphys.2019.01583

**Published:** 2020-01-22

**Authors:** Jugajyoti Baruah, Anju Vasudevan, Rüdiger Köhling

**Affiliations:** ^1^Department of Psychiatry, Harvard Medical School, Boston, MA, United States; ^2^Angiogenesis and Brain Development Laboratory, Division of Basic Neuroscience, McLean Hospital, Belmont, MA, United States; ^3^Oscar-Langendorff-Institute of Physiology, Rostock University Medical Center, Rostock, Germany

**Keywords:** vascular endothelia, development, blood-brain barrier, inflammation, hyperexcitability, epileptogenesis

## Abstract

Our understanding of the etiological mechanisms leading up to epilepsy has undergone radical changes over time due to more insights into the complexity of the disease. The traditional hypothesis emphasized network hyperexcitability and an imbalance of inhibition and excitation, eventually leading to seizures. In this context, the contribution of the vascular system, and particularly the interactions between blood vessels and neuronal tissue, came into focus only recently. Thus, one highly exciting causative or contributing factor of epileptogenesis is the disruption of the blood-brain barrier (BBB) in the context of not only posttraumatic epilepsy, but also other etiologies. This hypothesis is now recognized as a synergistic mechanism that can give rise to epilepsy, and BBB repair for restoration of cerebrovascular integrity is considered a therapeutic alternative. Endothelial cells lining the inner surface of blood vessels are an integral component of the BBB system. Sealed by tight junctions, they are crucial in maintaining homeostatic activities of the brain, as well as acting as an interface in the neurovascular unit. Additional potential vascular mechanisms such as inflammation, altered neurovascular coupling, or changes in blood flow that can modulate neuronal circuit activity have been implicated in epilepsy. Our own work has shown how intrinsic defects within endothelial cells from the earliest developmental time points, which preclude neuronal changes, can lead to vascular abnormalities and autonomously support the development of hyperexcitability and epileptiform activity. In this article, we review the importance of vascular integrity and signaling for network excitability and epilepsy by highlighting complementary basic and clinical research studies and by outlining possible novel therapeutic strategies.

## Introduction

**Figure 1:**
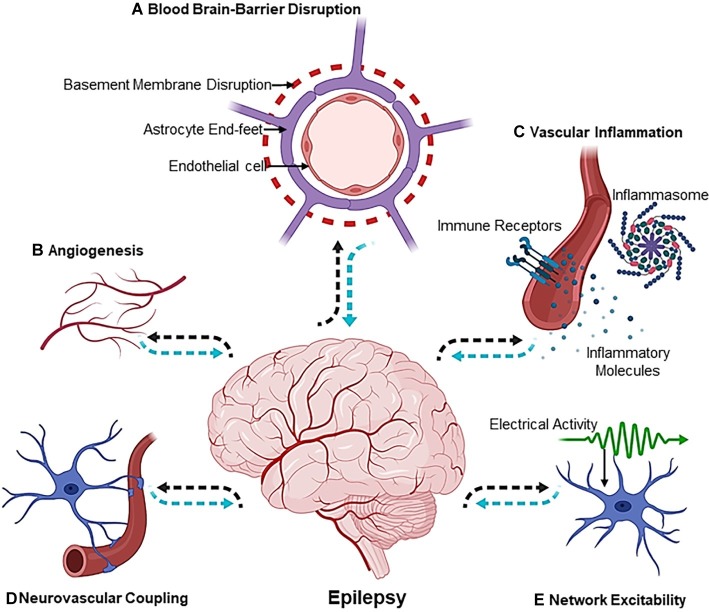
The Vascular Landscape in Epilepsy. The mechanisms involved in the etiology of epileptogenesis are multiphasic [**(A)** blood-brain-barrier disruption, **(B)** angiogenesis, **(C)** vascular inflammation, **(D)** neurovascular coupling and, lastly, **(E)** network excitability] and exist at the crossroads of the neurovascular network. Dotted black arrows indicate the sequence of events leading up to epileptogenesis, whereas dotted blue arrows show the sequence of events that affect the neuron or vascular interface in an epileptic brain. Illustration was created using Biorender.com.

Epileptogenesis is the process of developing epilepsy, that is, a chronic neurological disorder characterized by seizures. During this process, a normally functioning brain gradually develops the chronic susceptibility to generate intermittent or recurrent seizures. Epilepsy is a neurological disorder that not only impairs quality of life but also can lead to increased mortality (Devinsky et al., 2018; Beghi et al., 2019). Epileptogenesis can be precipitated by multifactorial events that can be either genetic or environmental. Nevertheless, the macro- or micro-molecular events that lead to epileptogenesis are poorly elucidated, and the disease mechanisms that lead to epilepsy are still unknown in 50% of the global cases (Neligan et al., 2012). Although extensive research using animal models of epilepsy has highlighted the wide array of molecular and cellular events that predict epileptogenesis (Avoli et al., 2005), the “missing link” between the primal events leading up to epileptogenesis in a human brain still persists. In an interesting editorial, a vivid interpretation of historical texts from the works of novelist and painter August Strindberg (1849–1912) was made. In his writings, he describes a person who, after several episodes with loss of tone and consciousness, shows a syndrome of complete hemiparesis due to stroke. This has been interpreted as *vascular precursor epilepsy* (Trinka et al., 2015), and therefore, it is safe to comprehend that the vascular component of epilepsy has been a topic of subtle discussion in history as well. However, it was not until the nineteenth century that an alternate hypothesis, which today is known as the “blood-brain barrier (BBB) hypothesis,” was proposed to explain some of the phenotypic consequences of epilepsy (Cornford and Oldendorf, 1986; Cornford, 1999). Studies from several other groups later added insights that directly implicated dysfunction in blood vessels to seizure disorders (Seiffert et al., 2004; van Vliet et al., 2006; Ivens et al., 2007; Marchi et al., 2007; Weissberg et al., 2011). In this context, our own work has depicted that selective deletion of vascular endothelial growth factor (VEGF), gamma aminobutyric acid (GABA) A receptor subunit beta 3 (GABRB3), or the vesicular GABA transporter (VGAT) from endothelial cells during early development affects forebrain vascular networks, leads to brain morphological defects, and makes lasting changes to cortical circuits (Li et al., 2013, 2018). Importantly, vascular health is of significance not only for epilepsy but also for several neuropsychiatric disorders (Baruah and Vasudevan, 2019). In Figure 1, we present a pictorial representation of “the vascular landscape in epilepsy,” highlighting different vascular or neurovascular abnormalities that are implicated in epilepsy through basic and clinical research. Many seminal reviews have addressed the role of BBB dysfunction in the etiology of epilepsy (van Vliet et al., 2006; Marchi et al., 2007, 2011; Kim et al., 2017), and therefore, the current review focuses on some of the studies in the last two decades and how information gained from these studies can be applied for novel therapeutic interventions.

## Vascular-Neuronal Interactions During Brain Development

The similarities between vascular and neuronal development in the brain are striking initially at a phenomenological level. In both the vascular and nervous systems, generation of the different types of cells begins with the proliferation of stem cells. Common mechanisms operate at the level of the cell cycle to regulate proliferation of angioblasts and neuronal precursors. In both, cell generation epochs result in an initial overproduction of cellular elements, and later, the excess elements are eliminated by apoptosis or pruning. Both vascular and neuronal elements undergo significant activity-dependent remodeling during development. In the case of the vasculature, the activity is generated by shearing force of blood flow, and in the case of the nervous system, the activity is generated by electrical impulses in the neuronal networks. Just as neuronal networks show plasticity in response to changing electrical activity, vascular endothelial cells also show remarkable plasticity in response to changing tissue oxygenation level or blood flow. Process outgrowth and guidance in the nervous system involve axon growth cone guidance, pathfinding, axon branching, and arborization. The corresponding events in the vascular system are filopodial extensions of tip cells (specialized endothelial cells at the growing tips of navigating vessels), migration of endothelial cells, vessel elongation, and sprouting. Thus, common mechanisms regulate the genesis of endothelial cells and neurons. A review of the literature indicates that not only an overlapping repertoire of signaling molecules but also intrinsic regulation by compartment-specific transcription factors controls angiogenesis, neurogenesis, and neuronal migration in the embryonic brain (Carmeliet, 2003; Haigh et al., 2003; Carmeliet and Tessier-Lavigne, 2005; Eichmann et al., 2005; Vasudevan et al., 2008; Li et al., 2013, 2018; Paredes et al., 2018; Karakatsani et al., 2019). Importantly, molecules produced in one system influence the other to promote proliferation, differentiation, migration, or process outgrowth in both the systems. This developmental phase is very crucial because improper neuro-vascular interactions can precipitate into a spectrum of neurological disorders including epilepsy (Carmeliet, 2005; Vasudevan and Bhide, 2008; Obermeier et al., 2013; Ruhrberg and Bautch, 2013; Stern, 2018). A common pathology seen in epileptic brains is the presence of widespread structural alterations in brain regions, such as the hippocampus, thalamus, or neocortex (Barkovich et al., 2015). Taken together, epilepsy can be characterized by shared disturbance in the cortico-subcortical brain network (Stafstrom and Carmant, 2015), and often times, it is concomitant with vascular malformation in young patients (Hauser and Mohr, 2011). Given that the blood vessels or the vasculature plays an important role in defining brain architecture and circuitry, any aberrant vascular-neuronal interactions during development stages when the brain is “wiring up” can be expected to make the brain more prone to develop epilepsy at postnatal stages or during adulthood.

## Abnormal Angiogenesis and Epileptogenesis

Angiogenesis is the spatio-temporal event where blood vessels are formed from preexisting vessels to perfuse tissues, establish circulation, and provide instructional cues both during development as well as in postnatal life as part of therapeutic angiogenesis. One of the earliest studies conducted by Rigau et al. showed an increase in angiogenic processes in the hippocampi that were surgically removed from patients with chronic intractable temporal lobe epilepsy (TLE; Rigau et al., 2007). An important set of molecules that regulates both developmental and pathological angiogenesis and is upregulated in patients with medically intractable epilepsy is the family of vascular endothelial growth factor (VEGF; Croll et al., 2004; Vezzani, 2005; Li et al., 2013; Sun et al., 2016). Interestingly, the subtypes A and B, and the VEGF receptors 1 and 2, were highly expressed in the dysplastic neurons. Since VEGF is expressed by both neuronal and endothelial cell populations, the authors concluded that VEGF-mediated signaling can act *via* autocrine or paracrine mechanisms that can lead to astroglial activation and precipitate events associated with epilepsy (Nicoletti et al., 2008, 2010; Li et al., 2013). The VEGF signaling pathway is also active in hippocampi, exhibiting epileptiform activity (Morin-Brureau et al., 2011). In a rat model of pilocarpine-induced epilepsy, there was an increased angiogenesis in the CA3 region of the hippocampus, which was also accompanied by an increase in cerebral blood flow. The increased angiogenic sprouting was accompanied by neurodegeneration, ectopic neurogenesis, mossy fiber sprouting in the hippocampus, and most importantly BBB leakage (Ndode-Ekane et al., 2010). In another rat model of mesial temporal lobe epilepsy, inhibition of angiogenesis *via* a chemical inhibitor, sunitinib, resulted in the absence of seizures when compared to sham-treated mice (Benini et al., 2016). Similar studies suggested that morphological changes observed in the epileptic foci were consistent with an increase in angiogenic processes. Most recently, patients who have mammalian target of rapamycin (mTOR)-dependent malformations of cortical development (MCDs) displayed hyperperfusion and an increased vessel density of the dysmorphic cortical tissue (Zhang et al., 2019). Ephrin receptor A4 that mediates neurogenesis and angiogenesis in the dentate gyrus was also found to increase angiogenesis in the CA1 and CA2 regions of the hippocampus in a mouse model of TLE (Feng et al., 2017). In another interesting study, astrocytes were shown to regulate angiogenesis *via* the activities of Jagged/Notch1 signaling pathway in a kainic acid-induced mouse model of epilepsy (Zhai et al., 2016). All of these studies have implicated abnormal angiogenesis in epilepsy.

## Developmental Angiogenesis and Origin of Epilepsy

A new perspective is that abnormalities in developmental angiogenesis can now be directly linked with the etiopathophysiology of epilepsy. Our studies have shown that during development, preformed vascular networks serve as a cellular substrate for long distance migration of GABAergic interneurons (Vasudevan et al., 2008; Won et al., 2013). Projection neuron precursors also interact closely with blood vessels during cerebral cortex development (Stubbs et al., 2009). Cortical abnormalities by virtue of defective migration and neuronal positioning are a commonly observed phenomenon in epileptic brains (Blinder et al., 2013; Whelan et al., 2018). Interestingly, the laminar position of cortical neuronal subsets has very little effect on the overall vascular pattern in the cortex in the shaking rat Kawasaki (SRK) and reeler mutants, despite inversion of cortical layers, suggesting the autonomous roles for vascular components in neurogenesis and neuronal migration (Vasudevan et al., 2008; Stubbs et al., 2009). Moreover, periventricular endothelial cells of the embryonic forebrain have a unique gene expression signature compared to pial endothelial cells or endothelial cells from the midbrain or hindbrain. Of significance, gene expression profiling depicted the disease category “epilepsy” to be significantly enriched in genes expressed in periventricular endothelial cells, whereas genes in pial endothelial cells showed enrichment in inflammation and pathological process categories. These observations implicate a new cell type, “periventricular endothelial cells,” as being contributory to epilepsy (Won et al., 2013).

**Figure 2:**
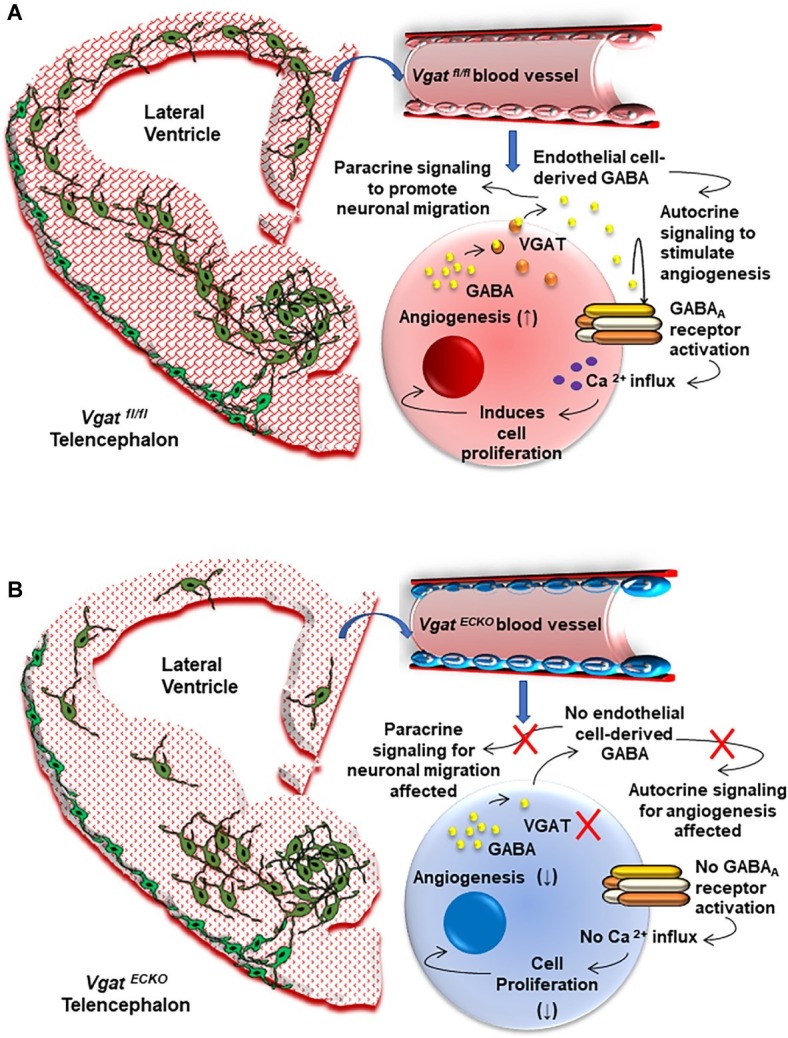
The Vascular Origin of Epilepsy. **(A)** Schema depicting the *Vgat ^fl/fl^* or control embryonic telencephalon at day 15 that has normal periventricular vascular network (red lattice pattern) and a normal endothelial GABA signaling pathway that promotes long distance tangential migration of GABAergic interneurons (green) from the ventral telencephalon. A single vessel has been magnified to illustrate the positive feedback GABA signaling pathway that exists in normal telencephalic endothelial cells (red). Endothelial GABA activates GABA_A_ receptors, thereby triggering Ca^2+^ influx and endothelial cell proliferation. Vesicular GABA transporter (VGAT) is the primary mechanism for GABA release from telencephalic endothelial cells at embryonic day 15 (Li et al., 2018). Endothelial GABA release thus has autocrine roles in stimulating angiogenesis (↑) and paracrine roles in promoting long distance GABAergic neuronal migration in the embryonic telencephalon. **(B)** Schema depicting altered vascular profiles in *Vgat ^ECKO^* telencephalon (red dotted pattern) in which there is complete loss of endothelial GABA secretion that causes GABAergic interneurons to stall in the ventral telencephalon. This has significant consequences for GABAergic neuronal tangential migration, resulting in neuronal reductions and abnormal cortical distribution in *Vgat ^ECKO^* telencephalon. Magnification of a single vessel to depict abnormal *Vgat ^ECKO^* endothelial cells (blue) and how lack of GABA release from these endothelial cells affects the positive feedback of GABA-GABA_A_ receptor signaling, which in turn significantly affects angiogenesis-related gene expression (↓). Lack of endothelial GABA release also affects paracrine signaling and impairs long-distance migration of GABAergic interneurons.

Defects in GABA_A_ receptor regulation or mutations in GABA_A_ receptor subunits and polymorphisms have also been associated with genetic epilepsies that cause seizures as one of the primary symptoms (Kang and Macdonald, 2009). In this respect, our work has unraveled a novel GABA_A_ receptor-GABA signaling pathway in periventricular endothelial cells of the embryonic brain that is distinct from the neuronal GABA signaling pathway. Selective deletion of the GABA_A_ receptor β3 subunit from endothelial cells resulted in seizure-like symptoms in 15% of the mice. On the other hand, deletion of the vesicular GABA transporter (*Vgat*) from endothelial cells resulted in a mouse model of epilepsy. We have shown that *Vgat* is the primary mechanism for GABA release from endothelial cells at early embryonic stages. Therefore, when *Vgat* was deleted specifically from endothelial cells, endothelial GABA secretion was turned off during embryonic brain development. In the absence of endothelial GABA release, all of the key cellular events during forebrain development – angiogenesis, neurogenesis, radial migration of projection neurons, and tangential migration of GABA interneurons – were affected to some degree, indicative of the autocrine and paracrine roles of EC-derived GABA signaling. Strikingly, gene expression profiling of *Vgat* endothelial cell conditional knockout (*Vgat ^ECKO^*) embryonic telencephalon at embryonic stages was able to predict the postnatal phenotype of the mouse model. Significant enrichment was seen in disease categories “epilepsy” and “seizures,” and several of the epilepsy-related genes were isolated and grouped (Li et al., 2018; Choi and Vasudevan, 2019). The *Vgat ^ECKO^* mice were smaller in size at birth when compared to littermate controls and developed severe seizures during the postnatal period P7–P14. Reduction in vascular densities was associated with a layer-specific loss of GABAergic interneurons along with abnormal GABAergic and glutamatergic neuronal distribution in *Vgat ^ECKO^* cerebral cortex indicative of a highly asynchronous cortical circuitry. *Vgat ^ECKO^* mice showed periods of quiescence, interrupted by tremors and reductions in movement, and did not survive beyond 2 months of age. Field potential recordings depicted a high degree of hyperexcitability in the hippocampus. Collectively, our work has provided mechanistic understanding of how intrinsic defects within blood vessels from the earliest developmental time points can directly contribute to epilepsy (Figure 2).

## Basement Membrane Dysfunction and Epilepsy

The basement membrane is a specialized form of extracellular matrix that encases the endothelial cells. Pericytes are also embedded in the basement membrane, situated between endothelial cells and astroglial endfeet. The composition and structure of the basement membrane affect the permeability of the vessel. Basement membrane constituents include collagens, laminins, fibronectin, fibrillin, vitronectin, perlecan, and nidogen, as well as growth factors and cytokines, enzymes responsible for matrix degradation, and proteins that adhere to the extracellular matrix, for instance, the semaphorins and lectins. Apart from providing a structural base for cells to adhere to, individual basement membrane components serve as regulators of many biological activities, such as cell growth, repair, differentiation, migration, proliferation, and morphogenesis. The effects of various basement membrane components on cell functions are mediated *via* cell surface receptors, such as integrins and dystroglycan. Mechanosensitive signals conveyed by the extracellular matrix have also been shown to converge with microenvironmental cues, such as growth factors (VEGF) to control transcription factors essential for angiogenesis (Mammoto et al., 2009). However, there is significant variability in the composition of basement membranes between small and large blood vessels throughout the brain. Targeted deletion of basement membrane components causes extensive cortical ectopias and dysplasia (Georges-Labouesse et al., 1998; Anton et al., 1999; Halfter et al., 2002). Although several neurological disturbances have been described as a result of basement membrane disruption, the knockout of nidogen-1 was the first report of a basement membrane gene that resulted in epilepsy. Interestingly, interference with nidogen altered neuronal excitability and synaptic plasticity without obvious underlying structural damage. Our work has shown that nidogen ablation leads to epileptic activity *in vivo* and the appearance of spontaneous epileptiform activity *in vitro* and opened the possibility of modulatory mechanisms of synaptic plasticity and excitability reaching beyond classical processes confined to cellular interactions (Köhling et al., 2006; Vasudevan et al., 2010).

Additionally, 8–20% of patients with laminin α2 deficiency suffer from seizures that arise in early childhood. Seizures were also observed in 8% patients with *LAMA2* mutations, suggesting that seizures may be present in a significant proportion of patients with primary laminin α2 mutations (Jones et al., 2001). Alterations in vasoregulation that included degeneration of pericytes, accompanied by abnormal basement membrane thickening in cerebral microvessels, have been reported in patients with intractable complex partial seizures (Liwnicz et al., 1990). Together, these studies establish contributions of basement membrane dysfunction in epilepsy.

## Blood-Brain Barrier Failure in Epileptogenesis

The BBB is a highly complex and dynamic structure that separates the circulating blood from the brain and is mainly composed of endothelial cells, pericytes, astrocytes, and the basement membrane. While an activated vascular system leads to altered angiogenesis, the common phenotype associated with this is an increase in barrier permeability or alteration of adherens junction protein that ultimately leads to disruption of the BBB system (David et al., 2009; Friedman, 2011; Abbott and Friedman, 2012; Bar-Klein et al., 2017; Stern, 2018). The disruption of the BBB can induce a multifaceted pathological process including but not limited to changes in the brain environment, altered neuroglial interactions, maladaptive angiogenesis, and hemodynamic changes in different brain regions. At the cellular level, the BBB is a micro-anatomic structure that mediates exchange of nutrients, xenobiotics, blood components, and cells, which is necessary for brain homeostatic functions (Obermeier et al., 2013). Given the proximity of the BBB microvessels to neurons, it is now increasingly accepted as a cause-effect factor in epilepsy, meaning that BBB failure may result in abnormal excitability of neurons or the neuronal network. Regarding the pathogenesis of epilepsy, after initial speculations on BBB failure leading to epilepsy in the 1960s (Quadbeck, 1968) and experimental studies in the 1970s confirming edema formation in focal experimental epilepsy (Nagy and Fischer, 1978), several seminal papers by the Friedman lab (Seiffert et al., 2004; Ivens et al., 2007; Bar-Klein et al., 2017; Lippmann et al., 2017) established a causality between BBB breakdown and epilepsy, mediated *via* albumin release into the brain parenchyma and subsequent TGF-β-dependent albumin glial uptake. The link between BBB disruption and epileptogenesis was consecutively confirmed in different models. Thus, recent evidence indicates that the dysfunctional BBB can (1) promote seizures, (2) contribute to epileptogenesis, and (3) favor seizure recurrence in patients with epilepsy (Daneman and Prat, 2015; Zhao et al., 2015; Rüber et al., 2018). One of the many proposed mechanisms by which BBB damage can lead to epileptogenesis is *via* a systemic intravascular inflammation (Vezzani and Friedman, 2011; Kim et al., 2017). In light of these findings, one can assume that BBB disruption is the pivotal event, which subsequently can lead to secondary events, such as altered neurovascular coupling (NVC), changes in the morphology of neurovascular network, and altered cerebral blood flow to different brain areas and systemic vascular inflammation (Figure 1).

The BBB is far from mature at birth. By embryonic day 15 (E15) in mice, the primitive BBB system is established *via* recruitment of pericytes to blood vessels, interaction of endothelial cells with the astrocytic endfeet, and further modifying the cell-cell junctions (Zhao et al., 2015). The BBB continues to mature after birth, although it is a species-specific temporal event (Daneman and Prat, 2015; Zhao et al., 2015). As the BBB continues to mature, improper establishment can lead to long-term implications in the brain and contribute to epileptogenesis. While a compromised BBB can have severe implications for the availability of glucose or drugs (Cornford and Oldendorf, 1986; Cornford, 1999), the experimental evidence cited above demonstrated direct seizure-promoting effects of a disrupted BBB. From a developmental standpoint, due to the fact that seizure activity is recorded in the postnatal stages, much of the current evidence is based on either animal or human systems analyzed at postnatal stages or during adulthood. One commonality observed in these cases was the presence of a leaky BBB system. A curious question is whether malformed BBB during development predicts epileptogenesis in a brain or whether ectopic events that result in a leaky BBB transform an otherwise healthy brain into an epileptic one? This remains an open-ended question, which Freidman famously stated as the “chicken and the egg puzzle” (Friedman, 2011). The above statement stems from experiments where either acute or chronic disruption of the endothelial tight junctions was induced that resulted in a hypersynchronous epileptiform activity (Seiffert et al., 2004; Marchi et al., 2007). Interestingly in our *Vgat ^ECKO^* model of epilepsy, we observed a reduction in tight junction proteins (claudin-5 and ZO1), IgG leakage, and increased vascular permeability at embryonic day 17 (Li et al., 2018). Endothelial GABA thus seems to have novel roles in strengthening tight junctions and is important for BBB development (Li et al., 2018).

The mechanisms by which a disrupted BBB in the affected brain can lead to epileptogenesis are multifold. One current hypothesis is that a leaky BBB can cause seizure-promoting components, such as albumin extravasation, excitatory glutamate neurotransmitter, and K^+^ ions to act on neuronal cells, which then increase network excitability (Ivens et al., 2007; Köhling and Wolfart, 2016). Pivotally, Ivens et al. found that a leaky BBB releases albumin that binds to TGF-β1 receptors on astrocytes and leads to astrocytic impairment of K^+^ spatial buffering and hence increased excitability. In a similar way, after BBB breakdown, glutamate uptake into astrocytes is compromised, thus possibly leading to a loss of excitability regulation as warranted by glutamate buffering (Heinemann et al., 2012). Apart from depolarizing neurons, higher levels of glutamate could also affect endothelial cells. Endothelial cells, including the neuronal cells, express the N-methyl-D-aspartate receptor (NMDAR), which is responsive to glutamate. A study conducted by Vazana et al. (2016) showed that glutamate can act on endothelial NMDAR, thereby inducing a leaky BBB; however, a separate commentary by Xhima et al. (2016) mentioned that since NMDARs are pantropic receptors expressed in the brain, the exclusive role of endothelial cells may not be central to the barrier disruption. In fact, the events might have resulted from an interplay of both vascular and neuronal NMDAR activities (Vazana et al., 2016; Xhima et al., 2016). In addition to endothelial cells, pericytes are also an integral component of the BBB system, and deficiency of pericytes is implicated in barrier disruption (Winkler et al., 2011). Pericytes along with vascular smooth muscle cells have been reported to play a central role in seizure-induced neurovascular remodeling, and these cells are added and removed from veins, arterioles, and capillaries after seizure induction with severe consequences for vessel physiology (Arango-Lievano et al., 2018). Another interesting aspect that is implicated in epilepsy and BBB breakdown, both in TLE patients and in animal experimental models of epilepsy, is the altered expression of aquaporins, the water channels responsible for maintaining fluid homeostasis (Binder et al., 2012; Heinemann et al., 2012). Lastly, also a reduction in GABAergic inhibition could be a factor of BBB breakdown, as demonstrated experimentally in peri-infarct hippocampal tissue (Lippmann et al., 2017).

## Neurovascular Coupling

The aspect of NVC is an emerging area in the etiology of epilepsy. NVC mechanism broadly entails the relationship between neuronal activity, tissue level oxygenation, and the flow of blood in the affected area (Schwartz, 2007). Cellular contributors to NVC in the neocortex include different types of neurons, endothelial cells, pericytes, vascular smooth muscle cells, and astrocytes. In an epileptic brain, this mechanism is compromised or uncoupled to meet the metabolic demands around epileptic focus and in deep cortical layers (Zhao et al., 2011). Prager et al. reported that seizure-induced injury to the microvasculature is associated with impaired NVC, which ultimately led to BBB dysfunction (Prager et al., 2019). Using a rat ictal model, Ma and colleagues demonstrated, in real time, the dynamics of NVC and uncoupling, during the initiation and events leading up to the termination of an ictal propagation (Ma et al., 2013). The use of voltage-sensitive dyes (VSDs) in their study helped image the cerebral blood flow (CBF) as well as changes in the membrane potential, indicative of ongoing coupling and uncoupling events in real time. Similarly, through the use of simultaneous three-dimensional (3-D) photoacoustic tomography and EEG, NVC events were validated in an animal model of epilepsy (Wang et al., 2014). Interestingly, in a case study of a patient with acute subarachnoid hemorrhage, events such as impaired NVC to ictal epileptic activity and spreading depolarization were seen. The authors posit that this might be a potential link to the BBB dysfunction (Winkler et al., 2012).

During epileptogenesis, in parallel to the vascular responses, neuronal responses also mediate blood vessel function, such as modulating blood flow or inducing angiogenic activities in the responding niche (Attwell et al., 2010). The two major signaling events involved in this process are the neuronal and the astrocytic signaling. Nitric oxide synthase containing neurons have been reported to participate in coupling between local cortical blood flow and synaptic signaling, a form of NVC that does not depend on metabolic needs (Estrada and DeFelipe, 1998). With respect to the neuronal signaling, NMDA or glutamate activates NMDA receptors on cortical neurons that trigger calcium influx, membrane depolarization, activation of intracellular nitric oxide synthase (neuronal; nNOS), and subsequent release of nitric oxide (Busija et al., 2007). This nitric oxide diffuses to cerebral arteries and arterioles and causes vasodilation, modulating the blood flow. The mechanism causes relaxation of the vascular smooth muscles without the involvement of astrocytes or endothelial cells. In astrocyte signaling, it is theorized that blood vessel dilation may occur through a K^+^-based mechanism *via* modulatory effects of oxygen (O_2_; Attwell et al., 2010).

GABAergic interneurons are also known to provide an exceptionally rich innervation to local microvessels and can transmute afferent neuronal signals to appropriate vascular responses, thus acting as local integrators of NVC (Vaucher et al., 2000; Cauli et al., 2004). Changes in blood flow have also been implicated in providing spatial and temporal information by modulating the neural activity and regulating the excitability of cortical circuits (Moore and Cao, 2008). Thus, a deeper understanding of hemodynamics and neural activity can be applied for elucidating vascular dysfunctions in epilepsy.

## Inflammation and Network Excitability

The mature vasculature and the mature nervous system can respond to infection or injury by activating defense mechanisms including inflammation. Another aspect in the development of epileptogenesis is inflammation in the brain that serves as an essential feature in hyperexcitable brain regions or tissues. Interestingly, endothelial cells are at the forefront of an inflammatory cascade. In this context, given the heterogeneity of the cerebral vasculature, pathological deviations from normal functions can directly impact neuronal firing events (Librizzi et al., 2018). Therefore, it is not surprising that an event that triggers seizure or epileptic activity in the brain will directly or indirectly affect the vasculature (Vezzani et al., 2010, 2013). Studies reveal that the inflammatory receptor TGF-β-mediated signaling cascade is one of the central processes that mediate key events associated with epileptogenesis (Cacheaux et al., 2009). *In vivo* studies utilizing a murine model of CD8 T-cell-mediated central nervous system inflammation demonstrated that neuronal VEGF is significantly upregulated, which is typical of neuroinflammation-induced BBB disruption (Suidan et al., 2010). While the group did not measure epileptic events in these mice, this study demonstrates the cellular interplay that can precipitate into epileptic events. Similarly, CCR5, a chemokine that regulates inflammatory processes was shown to control vascular inflammation and leukocyte recruitment during acute excitotoxic seizure induction and neural damage (Louboutin et al., 2011). Endothelial inflammasome molecules, such as NLRP1 and NLRP3, NOD1 and NOD2, and NLRC4 and NLRC5, have been implicated in brain injury processes, and these molecules can further our understanding of functional congruence at the vascular-neuron interface (Lénárt et al., 2016). Indeed, various neurological insults result in alterations of inflammatory mechanisms (Kim et al., 2017). The reports included in this review are not exhaustive. A similar correlation can be inferred from additional scientific evidences supported by either non-rodent or primate models of epilepsy. We have summarized some of the key studies in the epileptic patient population that were vascular centric in Table 1.

**Table 1:** Studies of interest in vascular-based human studies of epilepsy.

Method	Type of sample	Findings	References
Immunohistochemistry	Post mortem hippocampal sections	Altered microvascular network	Kastanauskaite et al. (2009) and Alonso-Nanclares and DeFelipe (2014)
ESI/MSI and BOLD	Patient population	Blood oxygenation level-dependent (BOLD) signal changes at the time of interictal epileptic discharges (IEDs) to identify their associated vascular/hemodynamic responses	Heers et al. (2014)
Sandwich enzyme-linked immunosorbent assay to determine Adhesion Molecules in CSF and sera	Patient group	Detection of soluble vascular cell adhesion molecule-1 (sVCAM-1) and soluble intercellular adhesion molecule-1 (sICAM-1) in sera, and CSF	Luo et al. (2014)
7T imaging	Patient group	Vascular dysgenesis implicated in the pathogenesis of polymicrogyria and epilepsy	De Ciantis et al. (2015)
Protein expression *via* Western Blotting	Patient group	Increased protein expression of VEGF-A, VEGF-B, VEGF-C, and their receptors in the temporal neocortex of pharmacoresistant temporal lobe epilepsy patients. Elevated expression of VEGF-C and its receptors, VEGFR-2 and VEGFR-3, in patients with mesial temporal lobe epilepsy	Castañeda-Cabral et al. (2019) Sun et al. (2016)
Neuropsychological assessment, clinical examination, and fasting blood evaluation for quantification of vascular status	Patient group	Aging persons with chronic epilepsy exhibit multiple abnormalities in metabolic, inflammatory, and vascular health that are associated with poorer cognitive function	Hermann et al. (2017)
Molecular and biochemical methods	Patient group	cDNA microarray showed the presence of CYP enzymes in isolated human primary brain endothelial cells	Ghosh et al. (2010)
Molecular and Biochemical methods	Post mortem and patient group	Endothelial cells of blood vessels are the major source of IL-17	He et al. (2016)
Immunohistochemistry	Patient group	Reduced ratio of afferent to total vascular density in mesial temporal sclerosis. Significant reduction in the density of afferent vessels	Mott et al. (2009)

*ESI/MSI, electric source imaging/magnetic source imaging; BOLD, blood-oxygen-level-dependent; VEGF, vascular endothelial growth factor receptor; CYP, cytochrome P-450*.

## Vascular Therapy for Epilepsy

Today, most of the pharmacological compounds available for epilepsy treatment are partially effective. One major reason is that the majority of people exhibit drug-resistant epilepsy (Walker and Köhling, 2013). An emerging consensus for this ineffectiveness of clinically available drugs is because majority of the drugs are designed in a way to target molecules and receptors specific to the neuronal cells. This is where we reiterate the need for pharmacologic compounds or biologics that can also target the non-neuronal cell population, such as the endothelial cells. Additionally, it is important to have a better understanding of cell type-specific contributions in epilepsy disease origin and a realization that it is not primarily a neuronal dysfunction. Remediating the BBB could be one option to prevent or shunt epileptogenesis (Shlosberg et al., 2010). Thus, BBB-affecting drugs such glucocorticosteroids have been used in children with difficult-to-treat epilepsies and were effective in reducing drug-resistant seizures and for restoration of BBB function (Marchi et al., 2012). Additionally, BBB-affecting drugs like natalizumab and IL-1RA act on proinflammatory mediators and have been tested in cases of refractory epilepsy, initially leading to suppression of seizures (Sotgiu et al., 2010), however, harboring the risk of severe side effects in the long run (Abkur et al., 2018). Thus, a search for less strongly interfering drugs is clearly necessary.

One possible intervention may be a modulation of mammalian target of rapamycin (mTOR). In neuron-specific (NS)-PTEN-depleted mice, progression into epileptogenesis was blocked by inhibiting mTOR signaling (Sunnen et al., 2011). Because aberrant mTOR signaling is concomitant with an increase in vessel density in epileptic patients, it will be interesting to see if targeting endothelial mTOR might alleviate or lead to a remission of epilepsy symptoms. In addition, an adjunct area that can be explored is the hormone therapy in epileptic patients. To this end, convincing data indicate that endothelial cells express estrogen or progesterone receptors, and both of these hormones have been shown to protect from vascular injury/BBB dysfunction in rodent models (Si et al., 2014; Shin et al., 2016; Yu et al., 2017). Therefore, targeting the vascular hormone receptors may serve as another approach to restore BBB defects, as well as for improving the angiogenic outcome in epileptogenesis. The vasoactive effects of soluble matrix proteins and integrin ligands may be tapped into in order to regulate calcium influx and modulate blood flow in arteries (Wu et al., 1998). For instance, identification of alterations in extracellular matrix-integrin signaling and treatment of epileptic mice with integrin blockers resulted in a significant reduction in kindling epileptogenesis (Wu et al., 2017). Another study has reported that loss of mural cells (that includes pericytes and vascular smooth muscle cells) is proportional to seizure severity and vascular pathology. Interestingly, intravenous treatment with platelet-derived growth factor subunits BB (PDGF-BB) activated PDGFRβ in mural cells, ameliorating vessel coverage with mural cells, vessel functions, and reducing spontaneous EEG epileptiform activity (Arango-Lievano et al., 2018). An added perspective in managing intractable epilepsy is introducing the ketogenic diet (KD). At least experimentally, KD may also positively affect astrocytic monocarboxylate transporters, and this change in turn in a recent study was associated with seizure reduction (Forero-Quintero et al., 2017). Since the molecular mechanisms of KD are far from understood, it would be interesting to understand the implication of KD on blood vessels, which can further our knowledge in designing effective diet-based interventions for epileptogenesis. While the list is not extensive, collectively, these studies bring to light the importance of vascular therapy in epilepsy.

In continuation, another experimental treatment for epilepsy is stem cell-based therapy. Human pluripotent stem cell (hPSC)-derived GABAergic interneurons can serve as a potential cell therapy for epilepsy because the therapeutic strategies are multiple: general secretion of GABA by the grafted cells to increase the seizure threshold, direct replacement of malfunctioning or lost GABAergic interneurons, or modulation of the excitatory hyperactive system (Cunningham et al., 2014; Zhu et al., 2018). However, one issue that needs improvement is the migration efficiency of transplanted cells. Transplanted human interneurons displayed minimal migration and distribution at 2 weeks posttransplantation. It was only at 4–7 months posttransplantation that migration and integration into the host brain were observed (Cunningham et al., 2014; Kim et al., 2014; Zhu et al., 2018; Upadhya et al., 2019). Therefore, the beneficial effects of interneuron graft-in-disease models were delayed to several months after transplantation. Another drawback is a reduction in GABA levels after transplantation that has been reported by some groups (Nolte et al., 2008; Alvarez Dolado and Broccoli, 2011). These are some current issues with moving this promising therapeutic treatment to the clinic. Vascular therapy could serve to improve this treatment strategy. Since the periventricular vascular network is the natural substrate for GABAergic neuronal migration in the embryonic forebrain, it can serve to improve hPSC-derived GABAergic neuronal migration. We have generated human periventricular-like endothelial cells in our laboratory using the hPSC technology that significantly improved the rate of human GABAergic neuronal migration after transplantation, with high GABA release levels (Datta et al., 2018). This endothelial-neuronal cotransplantation strategy may have significant benefits for brain repair in epilepsy.

## Conclusion and Perspectives

In this review, we attempt to shed light on how vascular health is crucial, if not one of the primary factors leading up to epileptogenesis. Greater challenges in the field are some of these lingering questions: (1) How to effectively target and prevent the brain from becoming hyperexcitable and seizure-prone after the first episode of seizure? (2) Which parameters in the blood vessel can help identify a brain that can become epileptic after ictal events? (3) Can we tap into the gene expression in embryonic forebrain blood vessels for better understanding of epilepsy? (4) Can we effectively use knowledge gained from developmental and vasculature-related studies to design interventions that will be effective in the clinic? These unmet questions warrant a deeper understanding and more research, necessitating new work highlighting vascular health. Molecular and mechanism-based studies can provide deeper understanding of pathways or genetic components involved in epileptogenesis. Translational studies or patient-based studies should be carefully evaluated to design targeted therapies in better management of this disease. Perhaps, fine tuning the vascular contribution or role in epileptogenesis can help find a “missing link” in epileptogenesis and will potentially serve as diagnostic or prognostic marker in years to come.

## Author Contributions

All authors listed have made a substantial, direct and intellectual contribution to the work, and approved it for publication.

### Conflict of Interest

The authors declare that the research was conducted in the absence of any commercial or financial relationships that could be construed as a potential conflict of interest.
